# Highly cooperative fluorescence switching of self-assembled squaraine dye at tunable threshold temperatures using thermosensitive nanovesicles for optical sensing and imaging

**DOI:** 10.1038/s41598-019-54418-1

**Published:** 2019-11-29

**Authors:** Keitaro Sou, Li Yan Chan, Satoshi Arai, Chi-Lik Ken Lee

**Affiliations:** 1 Research Institute for Science and Engineering, Waseda University, 3-4-1 Ohkubo, Shinjuku-ku, Tokyo 169-8555 Japan; 2 School of Mechanical & Aerospace Engineering, Nanyang Technological University, 50 Nanyang Avenue, Singapore, 639798 Singapore; 3 Department for Technology, Innovation and Enterprise, Singapore Polytechnic, 500 Dover Road, Singapore, 139651 Singapore; 4 WPI Nano Life Science Institute, Kanazawa University, Kakuma-machi, Kanazawa, 920-1192 Japan; 5 Division of Chemistry and Biological Chemistry, Nanyang Technological University, 21 Nanyang Link, Singapore, 637371 Singapore

**Keywords:** Materials chemistry, Chemistry, Materials science, Nanoscale materials, Nanoscience and technology, Nanoscale materials

## Abstract

Thermosensitive fluorescent dyes can convert thermal signals into optical signals as a molecular nanoprobe. These nanoprobes are playing an increasingly important part in optical temperature sensing and imaging at the nano- and microscale. However, the ability of a fluorescent dye itself has sensitivity and accuracy limitations. Here we present a molecular strategy based on self-assembly to overcome such limitations. We found that thermosensitive nanovesicles composed of lipids and a unique fluorescent dye exhibit fluorescence switching characteristics at a threshold temperature. The switch is rapid and reversible and has a high signal to background ratio (>60), and is also highly sensitive to temperature (10–22%/°C) around the threshold value. Furthermore, the threshold temperature at which fluorescence switching is induced, can be tuned according to the phase transition temperature of the lipid bilayer membrane forming the nanovesicles. Spectroscopic analysis indicated that the fluorescence switching is induced by the aggregation-caused quenching and disaggregation-induced emission of the fluorescent dye in a cooperative response to the thermotropic phase transition of the membrane. This mechanism presents a useful approach for chemical and material design to develop fluorescent nanomaterials with superior fluorescence sensitivity to thermal signals for optical temperature sensing and imaging at the nano- and microscales.

## Introduction

In recent years, numerous studies have focused on temperature at the nano- and microscale in the fields of chemistry, physics, biology, and nanotechnology. Several thermometric methods have been proposed for spatial measurements at the nano- and microscale. For instance, in nanoelectronics field, a system using a micro-cantilever allows for precise temperature measurements with superior spatial resolution on sample surfaces with an accuracy of milli-Kelvin order^1^. Nevertheless, contact thermometry, using a scanning probe, require long acquisition times and have limitations for measurements in wet samples and fluidic systems. Temperature sensing and imaging based on temperature-dependent fluorescent signals from a fluorescent reporter possess a high detection sensitivity and a superior temporal resolution; therefore, this is a promising and practical approach for thermometry at nano- and microscales in biology, nanobiotechnology, and nanomedicine fields^2–5^. This methodology simply requires a thermosensitive fluorescent material and an optical microscope or detector. Accordingly, many research groups have devoted their attention to the development of advanced fluorescent materials with superior performances for temperature sensing.

Various materials including fluorescent dyes^6–8^, fluorescent proteins^9^, upconverting nanoparticles^10^, polymer dots^11^, quantum dots^12^, nanodiamond^13^, gold nanoclusters^14^, and dye-embedded polymer nanoparticles^15,16^ and nanosheets^17^ have been proposed as fluorescent temperature sensing materials. Their working principle is based on temperature-dependent fluorescent signals, such as intensity, lifetime, and wavelength. Among these, intensity-based thermometry is preferred over lifetime and wavelength for high temporal resolution. The ability of intensity-based thermometers is determined by their sensitivity to temperature, which is evaluated from how much fluorescence intensity changes per every 1 °C^3,4^. Usually, the intensity of fluorescent materials linearly decreases with increasing temperature (sensitivity: 2–4%/°C). To improve the sensitivity, for example, fluorophores chemically conjugated with a thermosensitive polymer^18–20^ or DNA^21,22^ have been investigated. Conjugation increases sensitivity to ~7%/°C as the fluorescence intensity cooperatively changes with the thermosensitive polymeric or DNA matrix depending on the temperature. The design concept of the fluorescent dyes used in matrix systems is different from that of conventional thermosensitive fluorescent dyes because their fluorescence must cooperatively change with the temperature-sensitive matrices, rather than only their own sensitivity to temperature. Such approach, i.e., control the fluorescence emission characteristics of a dye with a temperature-sensitive matrix, enables the enhancement of sensitivity and the reduction of environmental noises beyond the ability of the fluorescent dye itself because of the cooperative effect of the matrix. Therefore, the high cooperativity of the matrix and the cooperative response of the fluorescent dye to the matrix are crucial for the creation of advanced fluorescent sensor materials.

Nanovesicles (NV) that form nano-sized capsule structures with lipid bilayer membranes have been used as biocompatible nanocarriers to deliver drugs to specific sites of action^23^. A typical thermal characteristic of these NV is the reversible phase transition at a specific temperature, called phase transition temperature^24^. Since the molecular packing state and the permeability of the lipid bilayer membrane cooperatively changes at the phase transition temperature, the phase transition of the lipid bilayer membrane is used for the release of the encapsulated cargo from the NV at a threshold temperature^25–29^. From the aspects of temperature sensing and imaging, the cooperative switching of fluorescence quenching and emission at the phase transition temperature of NV is an attractive methodology for sensing and imaging of threshold temperature, which can be tuned according to the phase transition temperature of the NV. However, the off/on fluorescence switching in response to phase transition of a lipid bilayer is challenging. In a previous study, we investigated threshold temperature sensing based on the release-induced fluorescence emission of calseine from thermosensitive NV encapsulating concentration-quenching calseine solution (50 mM) at the phase transition temperature^27^. This approach presents potential applications to track and record the region that becomes exposed above the threshold temperature in a three-dimensional matrix. However, this approach does not correspond to temperature dropping because the release-induced emission is a one-way process that responds to temperature rising. A reversible fluorescence switching mechanism is necessary to obtain an advanced temperature sensor that operates for both rise and decrease of temperature.

**Figure 1:**
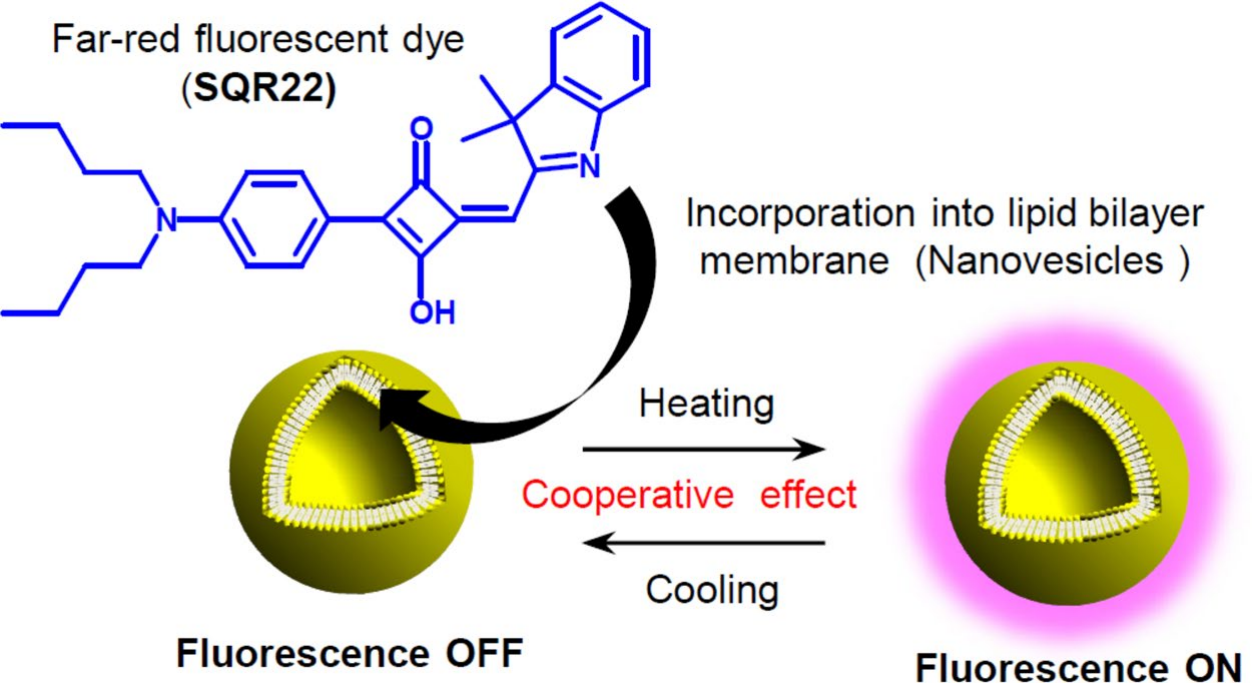
Fluorescence switching of self-assembled squaraine dye (SQR22) in lipid bilayer membrane of nanovesicles (NV) while heating and cooling.

Herein, we study a thermally reversible fluorescence switching of a unique squaraine dye incorporated into a self-assembled NV lipid bilayer membrane (Fig. 1). The results indicate the phase transition of the lipid bilayer membrane leads to aggregation and disaggregation of the fluorescent dye, which in turn induces the reversible fluorescence switching in a highly cooperative manner. This finding provides a useful and practical strategy based on molecular assembly control to create advanced fluorescent nanomaterials, which will further facilitate the development of temperature sensing and imaging at the nano- and microscales.

## Results and Discussion

### Spectroscopic properties of fluorescent dye SQR22

**Figure 2:**
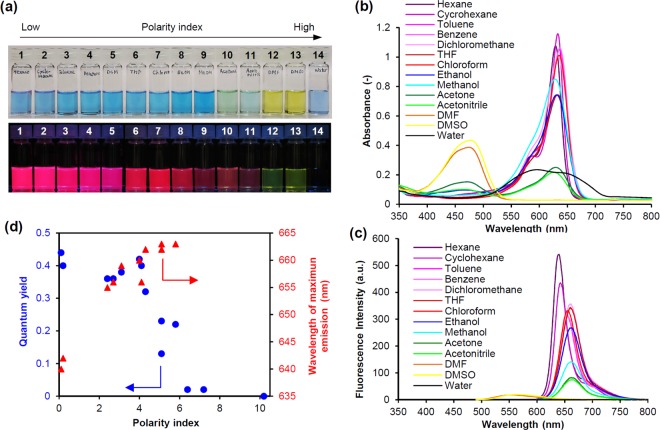
Spectroscopic properties of SQR22. (**a**) 10 µM SQR22 solutions in a bright field (top) and under UV irradiation (λ = 365 nm, bottom): 1 = hexane, 2 = cyclohexane, 3 = toluene, 4 = benzene, 5 = dichloromethane, 6 = tetrahydrofuran, 7 = chloroform, 8 = ethanol, 9 = methanol, 10 = acetone, 11 = acetonitrile, 12 = *N*, *N*-dimethylformamide (DMF), 13 = dimethyl sulfoxide (DMSO), 14 = water. (**b**) UV-vis-NIR spectra of 10 µM SQR22 solutions. (**c**) Fluorescence emission spectra of 1 µM SQR22 solutions. (**d**) Quantum yield and maximum emission wavelength as a function of polarity index.

An asymmetrical squaraine dye (SQR22) with dibutylaniline, squaric core, and indolium fragments was synthesized to study its properties as a far-red amphiphilic fluorescent probe (Fig. 1). A previous study showed that dibutylaniline is an important moiety in asymmetrical squaraine dyes that contributes to a significantly large Stokes shift^30^. Large Stokes shift of fluorescent dyes confers them advantages in fluorescence labeling applications because it reduces self-quenching effects and interference from the excitation source. First, we investigated basic spectroscopic properties of the dye in solution. The amphiphilic asymmetrical structure of SQR22 conferred it a good solubility in a wide range of solvents with varying polarity. The changes in color of the SQR22 solutions clearly indicated that the dye is affected by the polarity of the solvents (Fig. 2a). The polarity of the ground and excited states of the dye are different, hence, the stabilization of these two states is different and depends on the polarity of the solvent, which leads to a change in the energetic gap between these electronic states. Accordingly, the variation of the absorption and emission peaks and their intensity is a solvatochromic property^31,32^. Dyes with such characteristic can function as fluorescence probes to evaluate the environmental conditions in which the dye exists^33,34^.

UV-vis-NIR spectra of SQR22 solution showed the maximum absorption around 630 nm in polar solvents (Fig. 2b and Table S1). With increasing solvent polarity, the absorption around 630 nm decreased and an absorption peak appeared around 470 nm. In water, a broad absorption from 500 to 700 nm was observed. The fluorescence emission spectra generally showed that the wavelength of maximum emission gradually shifted from 640 to 663 nm in the far-red region with increasing solvent polarity (Fig. 2c,d and Table S1). The quantum yield was in between 0.13 and 0.44 in this region (Fig. 2d and Table S1). The wavelength of maximum fluorescence emission was 552 and 565 nm in DMF and DMSO, with a 0.02 quantum yield. Spectroscopic data is affected by several factors such as the aggregation state of organic dyes^35,36^, the equilibrium of protonation and deprotonation of dyes having dissociation group depending on environmental proton ion concentration^37^, as well as the solvatochromic property depending on the polarity of the solvent. The shift in the absorption spectra and lower quantum yield of SQR22 in DMF and DMSO could be due to multiple factors including solvatochromic property depending on the polarity of the solvent and the formation of aggregates depending on the solubility in the solvent. Since the solid of SQR22 could not be dissolved in water, it was dispersed in water by ethanol injection. In water, no fluorescence emission was observed probably because of self-aggregation.

Spectroscopic data indicated that SQR22 emits intense fluorescence at the far-red region (640–700 nm) with an excitation wavelength between 550 and 640 nm in nonpolar solvents. This excitation wavelength is adequate for various applications, such as fluorescence imaging, because the commercially available standard red laser is within this range (around 630 nm). The high quantum yield in solvents with low polarity index suggested that SQR22 is useful to label nonpolar environments. Therefore, SQR22 was expected to act as a potent far-red fluorescent probe to label the hydrophobic nonpolar region of a lipid bilayer membrane.

### Incorporation of SQR22 into a nanovesicles’ lipid bilayer

**Figure 3:**
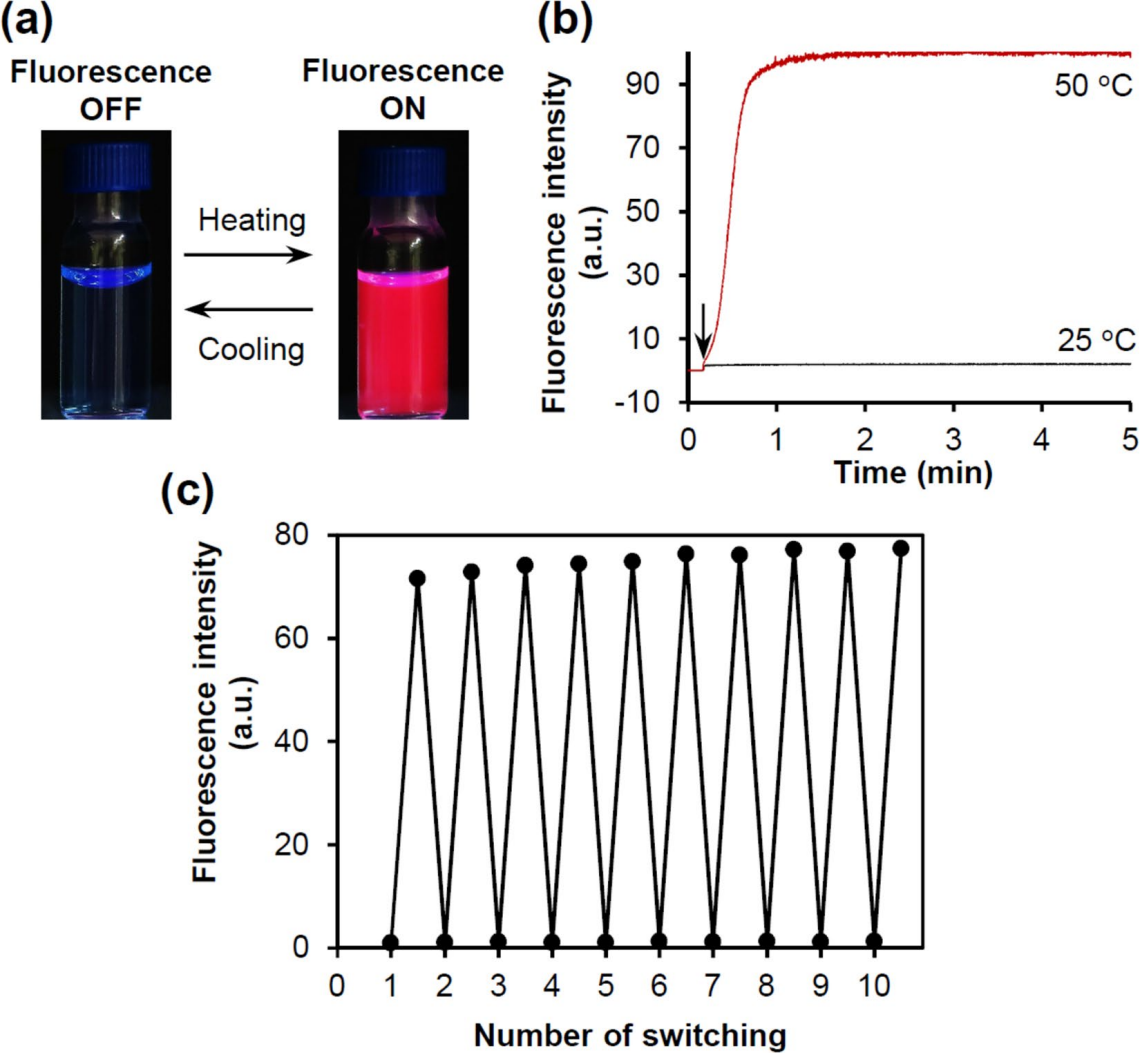
Reversible fluorescence switching on NV containing SQR22 (NVSQ) during heating and cooling. (**a**) Photographs showing the fluorescence of the NV consisting of a 1,2-dipalmitoyl-*sn*-glycero-3-phosphocholine (PC_16_) dispersion (PC_16_-NVSQ, Lipid/SQR22 = 50 w/w, [SQR22] = 10 µM)) under UV radiation (λ = 365 nm) at 25 °C (fluorescence OFF) and around 50 °C (fluorescence ON). (**b**) Profiles of fluorescence intensity changes upon heating of PC_16_-NVSQ at 25 and 50 °C. The arrow indicates the sample (*ca*. 23 °C) injection point into a quartz cuvette incubated at the target temperature. (**c**) Repeatability of fluorescence switching for PC_16_-NVSQ ([SQR22] = 1 µM) through 10 heating/cooling cycles. The fluorescence intensity was measured alternately at 25 (cooling) and 48 °C (heating).

Initially, a freeze-dried PC_16_ powder containing SQR22 (lipid/SQR22 = 50/1, w/w) was hydrated to obtain nanovesicles (NV) that incorporated SQR22 in the hydrophobic nonpolar domain of a lipid bilayer membrane (PC_16_-NVSQ). Unexpectedly, when SQR22 was in the nanovesicles, its fluorescence emission was not observed under UV radiation (λ = 365 nm) at around 25 °C. However, intense emission was observed when heating (Fig. 3a). Then, after cooling, the fluorescence disappeared again. As shown in Fig. 3b, fluorescence intensity quickly responded to the temperature changes. The initial rate constants of increasing fluorescence intensity were 7.0%s^−1^ at 50 °C. The actual response might be faster than the observed data because this measurement involves the time required to reach the target temperature. Fluorescence switching during the heating/cooling process was stable for at least 10 cycles (Fig. 3c). It is worth noting that fluorescence of SQR22 in organic solvents did not show considerable temperature dependency.

**Figure 4:**
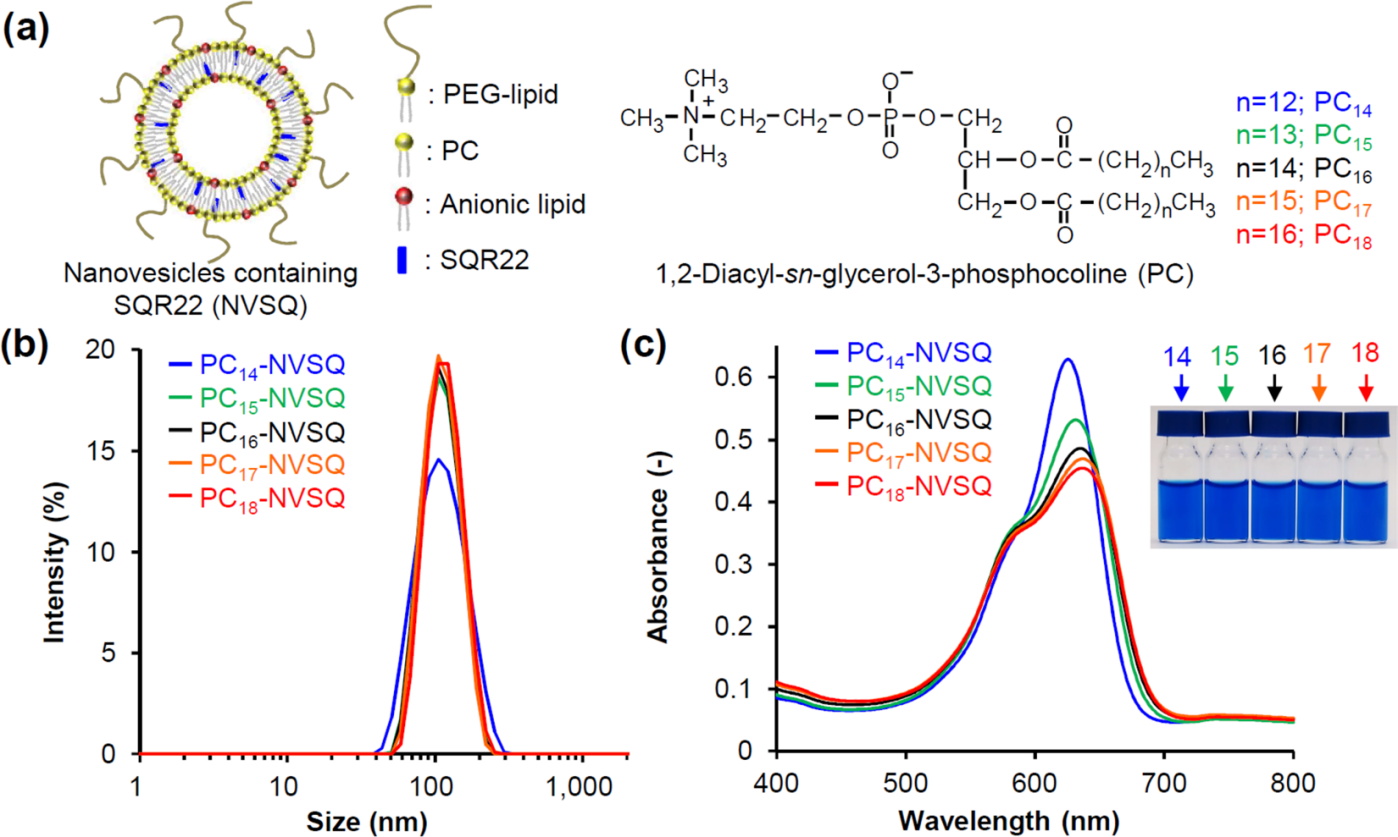
Incorporation of SQR22 into nanovesicles’ lipid bilayer membrane. (**a**) Scheme of the nanovesicles consisting of SQR22, 1,2-diacyl-*sn*-glycero-3-phosphocholine (PC), anionic lipid, and PEG-lipid (PC-NVSQ). Five PCs with different acyl chain lengths were used. (**b**) Size distribution of PC-NVSQ measured by dynamic light scattering. (**c**) UV-vis-NIR absorbance spectra of PC-NVSQ in phosphate buffered saline (PBS) at 25 °C ([SQR22] = 10 µM). The right photograph shows the PC-NVSQ dispersion ([SQR22] = 80 µM).

Based on these observations, we hypothesized that the fluorescence emission behavior of SQR22 in the lipid bilayer membrane switches with the phase transition of the membrane. To assess this, five kinds of 1,2-diacyl-*sn*-glycero-3-phosphocholine (PC) with increasing acyl chain lengths (14 to 18 carbon atoms) were used to prepare lipid NV containing SQR22 (PC-NVSQ) with different phase transition temperatures. Additionally, anionic (SA) and poly(ethyleneglycol) conjugated (PEG-lipid) lipids were incorporated to achieve a stable unilamellar vesicle dispersion (Fig. 4a)^38,39^. The hydrates of lipids and SQR22 were extruded through membrane filters (final pore size: 50 nm ϕ) to control the particle size. The size of the obtained PC-NVSQ was around 100 nm, independent of the lipid components (Fig. 4b and Table S2). The PC-NVSQ dispersion was blue with an absorption peak around 630 nm (Fig. 4c and Table S2), similar to the absorption peak of SQR22 in nonpolar solvents (Fig. 2b). This indicates that SQR22 was located in the nonpolar environment inside the lipid bilayer membrane. The maximum absorbance (λ_max_) shifted to longer wavelengths with increasing acyl chain length. Spectroscopic data reflects the aggregation state of organic dyes; aggregates that exhibit J- (bathochromic shift) or H-bands (hypsochromic shift) with respect to the monomer are known as J- or H-aggregates^35^. This bathochromic shift suggests the dyes forms J-aggregates. In addition, there is a shoulder peak at around 580 nm for the main peak of all PC-NVSQ. The hypsochromic shift of this peak is ascribed to the formation of H-aggregates. Thus, the absorption spectra suggested that SQR22 self-aggregates in the lipid bilayer membrane at 25 °C.

### Temperature dependence of the PC-NVSQ fluorescence emission

**Figure 5:**
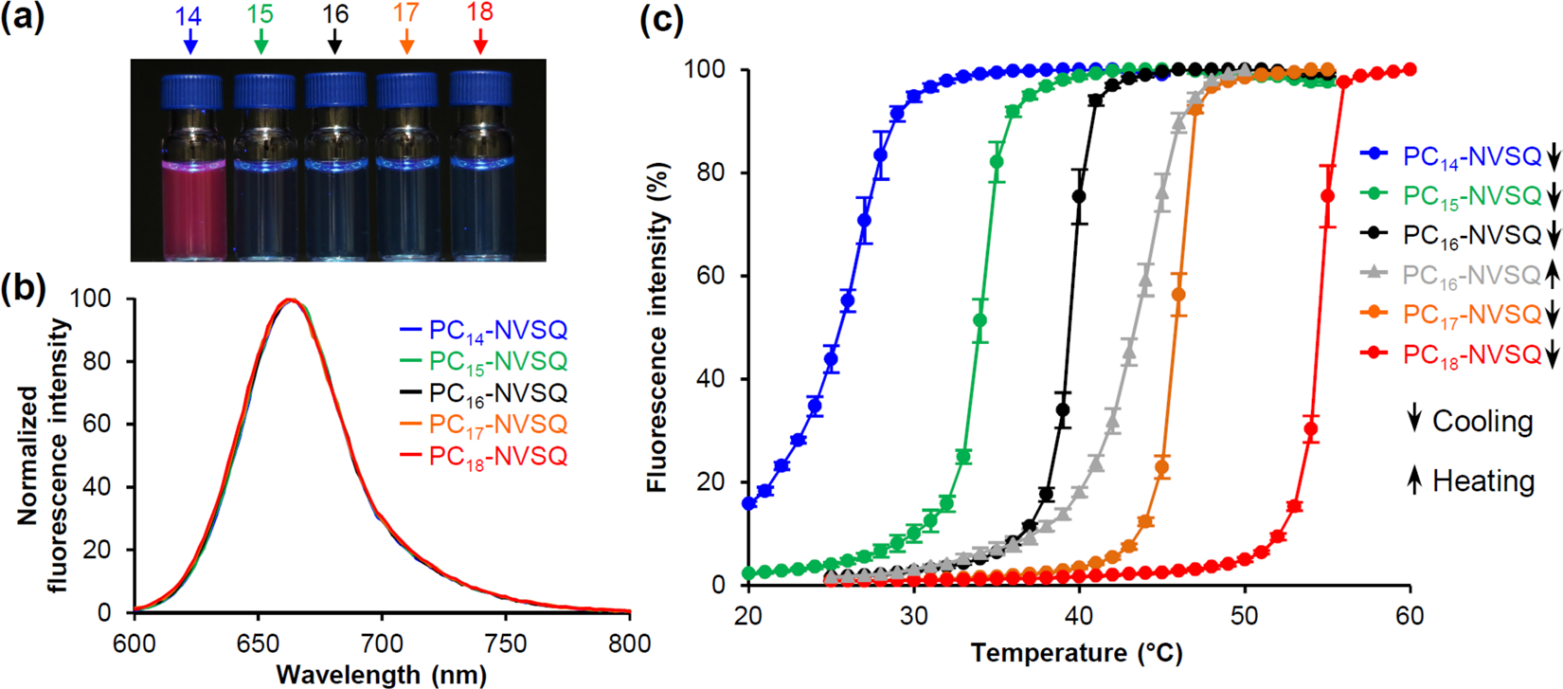
Temperature-dependent fluorescence characteristics of PC-NVSQ. (**a**) PC-NVSQ dispersions ([SQR22] = 10 µM) under UV radiation (λ = 365 nm) at 25 °C. Samples correspond to PC_14_-, PC_15_-, PC_16_-, PC_17_-, and PC_18_-NVSQ. (**b**) Fluorescence emission spectra of PC-NVSQ dispersion (Lipid/SQR22 = 50 w/w) at 40, 45, 50, 55, and 60 °C, respectively for PC_14_-, PC_15_-, PC_16_-, PC_17_-, and PC_18_-NVSQ (λ_ex_ = 570 nm). (**c**) Change in PC-NVSQ fluorescence intensity as a function of temperature ([SQR22] = 1 µM) during heating and cooling. Data are mean ± standard deviation (SD) acquired from three independent experiments.

We investigated the fluorescence properties of the five PC-NVSQ (lipid/SQR22 = 50/1, w/w). As expected, fluorescence was not observed under UV radiation (λ = 365 nm) at around 25 °C, except for PC_14_-NVSQ that has a phase transition temperature of 24 °C (Fig. 5a). When heating, all PC-NVSQ dispersions emitted intense fluorescence; their spectra were similar regardless of the host lipid NV (Fig. 5b). When heating, the emission peaks (λ_em_) were 664 nm for PC_14_-, PC_15_-, PC_16_-, and PC_17_-NVSQ, and 662 nm for PC_18_-NVSQ. Based on the correlation between maximum emission wavelength and polarity index (Fig. 2d), the polarity around SQR22 in the lipid bilayer membrane is comparable to that of solvents with 4.3–5.8 index, i.e., ethanol, methanol, acetone, and acetonitrile. In a previous study that uses a solvatochromic fluorescence cholesterol conjugate, *N*-(2-naphthyl)-23,24-dinor-5-cholen-22-amin-3β-ol, the polarity of the hydrocarbon region of phosphocholine bilayers was estimated to be similar to that of acetonitrile (polarity index = 5.8, dielectric constant (*ɛ*) = 37.5)^33^. Thus, our results agree with previous estimations, further supporting that SQR22 is located at the nonpolar region of the lipid bilayer membrane. It is likely that a highly-dynamic polarity gradient exists across the bilayer membrane, and that it is affected by the membrane charge and lateral pressure profile. In bilayer membranes, the polarity gradually decreases across the choline, phosphate, glycerol, and carbonyls, reaching a *ɛ* of approximately 2 in the hydrocarbon region; for comparison, the *ɛ* of bulk water is 78.5^40,41^. The relatively high polarity, experimentally estimated from the solvatochromic fluorescence, suggests that the chromophore of SQR22 is located around the glycerol backbone of PC (*ɛ* ~ 40) rather than the terminal methyl group of the hydrocarbon chains during heating^41^.

Next, the temperature dependence of the fluorescence emission of all PC-NVSQ was tested upon cooling (transition from liquid crystalline to gel state) to analyze the correlation between fluorescence intensity and the membrane’s phase transition temperature. In all cases, intensity decreased with decreasing temperature, showing typical sigmoidal curves. The onset temperatures to induce the steep fluorescence decrease were 29 ± 1, 36 ± 1, 41 ± 1, 47 ± 1, and 55 ± 1 °C, respectively, for PC_14_-, PC_15_-, PC_16_-, PC_17_-, and PC_18_-NVSQ (Fig. 5c). The corresponding phase transition temperatures are, respectively, 24, 35, 40, 49, and 55 °C (Table S2). It can be observed that these onset temperatures agreed with the phase transition temperature of the matrices. For PC_14_-NVSQ, the cooperativity of the sigmoidal curve was low and the flexion point of the curve (26 ± 0.2 °C) was close to the phase transition temperature.

Furthermore, PC_16_-NVSQ were tested during heating (transition from gel to liquid crystalline state). The onset temperature where a steep fluorescence intensity increase was induced upon heating was around 40 ± 1 °C, which agrees with the phase transition temperature of PC_16_-vesicles (40 °C). Thus, during both heating and cooling, the onset temperature fluorescence intensity changes were induced correspond to the phase transition temperature of the lipid NV. The fluorescence intensity-temperature profiles exhibited hysteresis, characteristic of first-order phase transitions. The hysteresis behavior on phase transition of PC_16_ (DPPC) bilayer membranes has been observed by differential scanning calorimetry, with a quartz crystal microbalance, and by theoretical simulation^42–44^. Hence, the fluorescence intensity changes of SQR22 reflect the phase transition of the specific lipid bilayer membrane during heating and cooling, which confirms our hypothesis. The fluorescence switching in the heating/cooling process was stable for at least 10 cycles for all PC-NVSQ (Figs. 3c and S4).

As previously reported, the ability of intensity-based temperature sensing is evaluated by the fluorescence intensity change for every 1 °C^3,4^. The sensitivity of fluorescent molecules used for thermometry is in range of 2–4%/°C^7^. In contrast, the average percentages of fluorescence intensity change around a flexion point of the sigmoidal curves shown in Fig. 5c are 10.0%/°C for PC_14_-NVSQ (28–22 °C), 19.0%/°C for PC_15_-NVSQ (36–32 °C), 20.6%/°C for PC_16_-NVSQ (41–37 °C), 21.2%/°C for PC_17_-NVSQ (47–43 °C), and 22.0%/°C for PC_18_-NVSQ (56–52 °C) during cooling, and 13.2%/°C for PC_16_-NVSQ (41–46 °C) when heating. These sensitivity values are significantly higher than that of conventional thermosensitive fluorescent nanoprobes. It has been demonstrated that the phase transition temperature of a fully hydrated membrane of saturated diacyl phosphocholine (PC) linearly correlates with the reciprocal of hydrocarbon chain length^24,29^. Thereby, the fluorescence switching temperature of PC-NVSQ can be readily tuned by changing the phase transition temperature, which is determined by the host lipid membrane. The increase or decrease in fluorescence intensity upon heating or cooling indicate that the local temperature around PC-NVSQ reached the threshold temperature, i.e., phase transition temperature of the lipid bilayer membrane. Thus, thermal sensing and imaging for such programed temperature could be easily accomplished by using a fluorescence microscope.

### Microscopic observation of fluorescence switching

**Figure 6:**
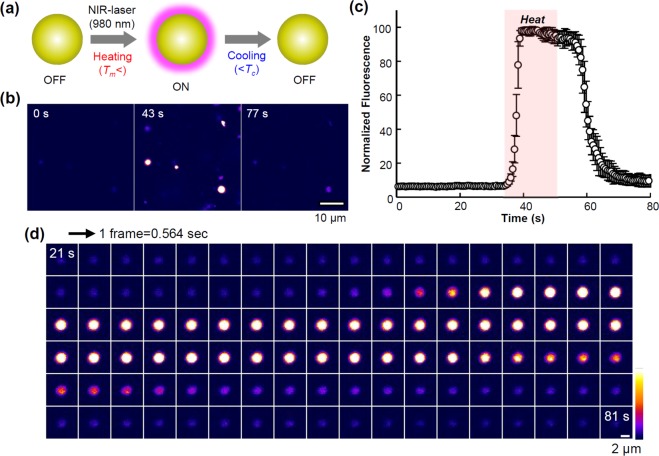
Microscopic monitoring of the reversible switching of fluorescence on PC_16_- multilamellar vesicles (MLV) containing 3.3 mol% SQR22 upon heating and cooling. (**a**) Schematics of fluorescence switching on vesicles containing SQR22 by heating with 980 nm NIR laser irradiation. (**b**) Fluorescence images of MLV containing SQR22 fixed in agarose gel; before heating (0 s), during heating (43 s), and after cooling (77 s). (**c**) Evolution of the SQR22 fluorescence emission. The fluorescence intensity is normalized to the maximum value. Average of the normalized intensity (four dots shown in **b**) was plotted against time. (**d**) Time-lapse images of MLV containing SQR22 as a “single dot” during the heating/cooling process. The single dot was chosen from those in (**b**).

We examined the fluorescence switching of PC_16_-NVSQ (switching temperature: 40–41 °C) to assess the feasibility of temperature sensing and imaging using a fluorescence microscope. Due to the spatial resolution limitations of the optical microscope, this experiment was conducted with PC_16_-multilamellar vesicles with the same composition than PC_16_-NVSQ. A near infrared (NIR)-laser (980 nm) was used to heat water by photothermal conversion^26^. Fluorescence was observed during heating with the laser irradiation and while cooling after irradiation (Fig. 6). As shown in Fig. 6b–d, the change in fluorescence intensity was negligible before irradiation. Then, it rapidly increased (ca. 40%s^−1^) when laser irradiation started, reaching an emission 15 times more intense than that before irradiation. Such an increase indicates that the observed area is heated to a temperature higher than the phase transition of the lipid membrane. Once irradiation stops, fluorescence decreases during cooling. This demonstrated that the fluorescence switching system is a promising tool for thermal imaging. As shown in Fig. 5c, the profile of fluorescence intensity with temperature exhibits hysteresis upon heating and cooling. This may complicate the correlation between fluorescence intensity and temperature. Further investigations on the mechanism of fluorescence switching are required to verify the feasibility of this system for thermometry.

### Mechanism of fluorescence switching at phase transition temperature

**Figure 7:**
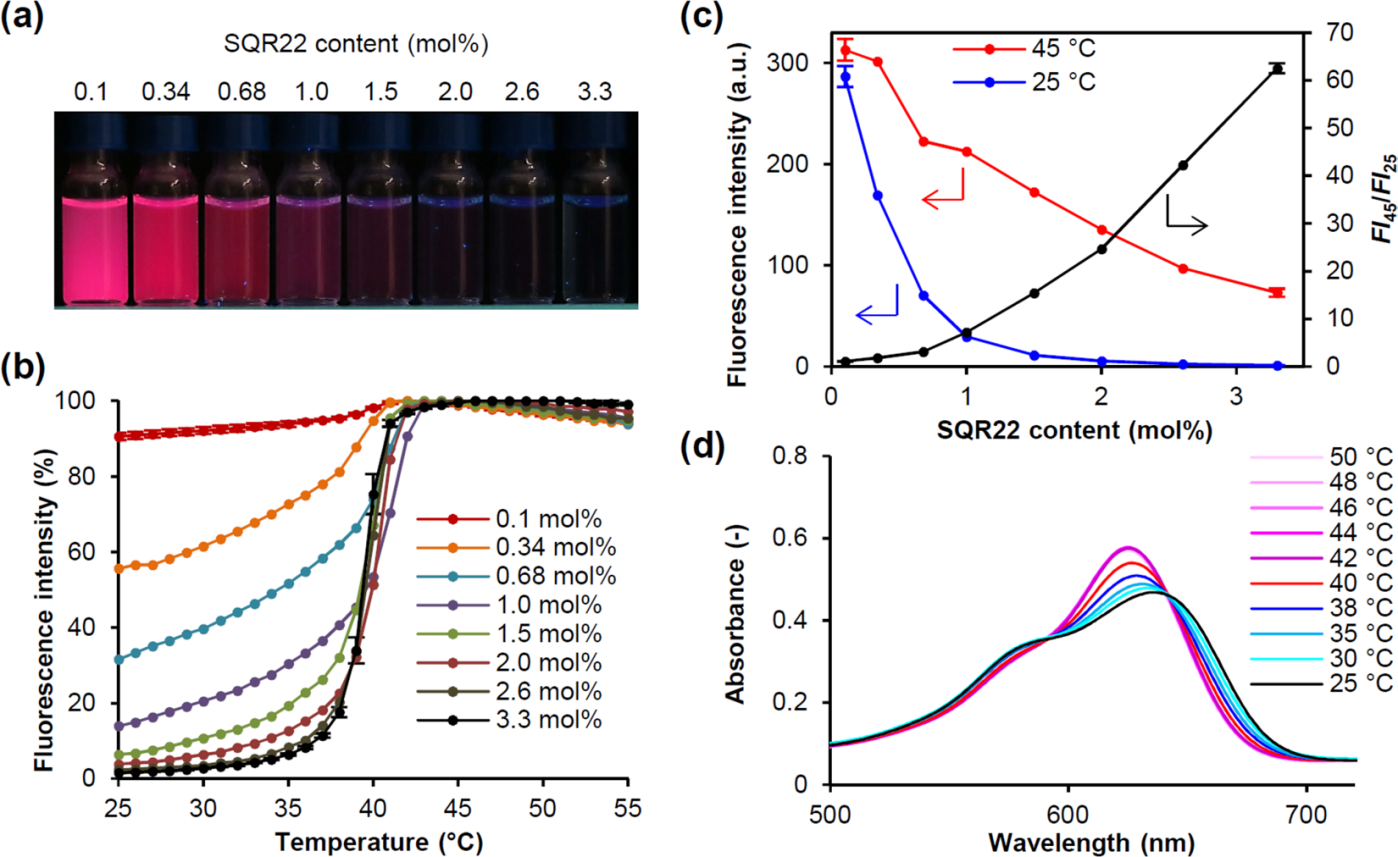
Spectroscopic analysis of fluorescence switching. (**a**) PC_16_-NV with different various amounts of SQR22 ([SQR22] = 10 µM) under UV radiation (λ = 365 nm) at 25 °C. (**b**) Change in fluorescence intensity of SQR22-loaded PC_16_-NV dispersions as a function of temperature. Data were acquired upon cooling. (**c**) Change of fluorescence intensity at 25 and 45 °C, and ratio of intensity at 25 (*FI*_25_) and 45 °C (*FI*_45_) as a function of SQR22 content in PC_16_-NV ([SQR22] = 1 µM). (**d**) Change in UV-vis-NIR spectra as a function of temperature in PC_16_-NV containing 3.3 mol% SQR22 ([SQR22] = 10 µM).

Based on the spectroscopic data shown in Fig. 4c, we hypothesized that the fluorescence of SQR22 is quenched due to self-aggregation in the lipid bilayer membrane in the gel state. Our previous study showed that a squaraine dye analog (SQR23) with dihexylaniline emits intense fluorescence at 0.1 mol% in a PC_16_ bilayer membrane, which is useful for bioimaging^45^. In contrast, the PC-NVSQ used in this study contains more than 3 mol% of SQR22. This suggests that the content of dye might be a critical factor for the quenching. Because self-assembly that leads to aggregation is a spontaneous equilibrium process between the aggregate and monomer, the ratio of SQR22 monomer, which emits fluorescence, should increase when decreasing its content in the lipid membrane. To verify this, the fluorescence properties of PC_16_-NV with different amounts of SQR22 were studied. As expected, fluorescence increased when decreasing the content of SQR22 in NV at around 25 °C, Fig. 7a. The degree of fluorescence intensity variation with temperature decreases when decreasing the SQR22 content (Fig. 7b). Finally, the temperature-dependent fluorescence change is minimal at 0.1 mol% of SQR22. This is likely caused by the shift in equilibrium between dissociated and aggregated SQR22 toward the dissociated fluorescing form when decreasing the amount of SQR22 in the lipid bilayer membrane. Temperature-dependence of fluorescence emission spectra of PC_16_-NV with 3.3 mol% SQR22 indicated that the fluorescence switching of SQR22 in lipid bilayer membrane at the phase transition temperature was not accompanied by a shift in the wavelength of maximum emission (Fig. S5). Since the solvatochromic properties of SQR22 induce the shift of emission peak as shown in Fig. 2, this result support that the involvement of the change in environmental polarity is minimal in the fluorescence switching of the dye in lipid bilayer membrane at the phase transition temperature. These results strongly support that the fluorescence switching of SQR22 is induced by aggregation-related photophysical properties of the dyes in lipid bilayer membrane without change in electronic state of SQR22.

The plots of fluorescence intensity at 25 °C and 45 °C show that it decreases with increasing SQR22 in both cases, despite the fact that the host bilayer membrane is in gel phase at 25 °C and liquid crystalline state at 45 °C (Fig. 7c). This indicates that fluorescence is partly quenched at higher SRQ22 content, even when the bilayer membrane is at the liquid crystalline state. However, when increasing the SQR22 content, the difference in intensity at 25 and 45 °C was increasingly larger because of the stronger quenching in the solid gel phase. The fluorescence intensity ratio between 45 (*FI*_45_) and 25 °C (*FI*_25_) was 1.1 ± 0.01 at 0.1 mol% and increased to 62.5 ± 1.03 at 3.3 mol% of SQR22. This is an attractive feature for the development of fluorescent temperature sensors with higher sensitivity.

UV-vis spectra of PC_16_-NV with 3.3 mol% SQR22 further supported the aggregation of SQR22 in lipid bilayer membranes at the solid gel phase (Fig. 7d). The absorption peak of 3.3 mol% SQR22 in PC_16_-NV was 636 nm at 25 °C. With increasing temperature, the absorption peak shifted to shorter wavelengths, reaching 625 nm at 42 °C. The bathochromic shift at lower temperatures is characteristic of J-aggregates. The shorter-wavelength shifted shoulder peak at around 580 nm (due to H-aggregates)^46^ decreased when the temperature increases above 40 °C. In contrast, the absorption peak of 0.1 mol% SQR22 in PC_16_-NV was 628 nm at 25 °C and the changes were less remarkable than those of 3.3 mol% SQR22 PC_16_-NV (Fig. S6).

**Figure 8:**
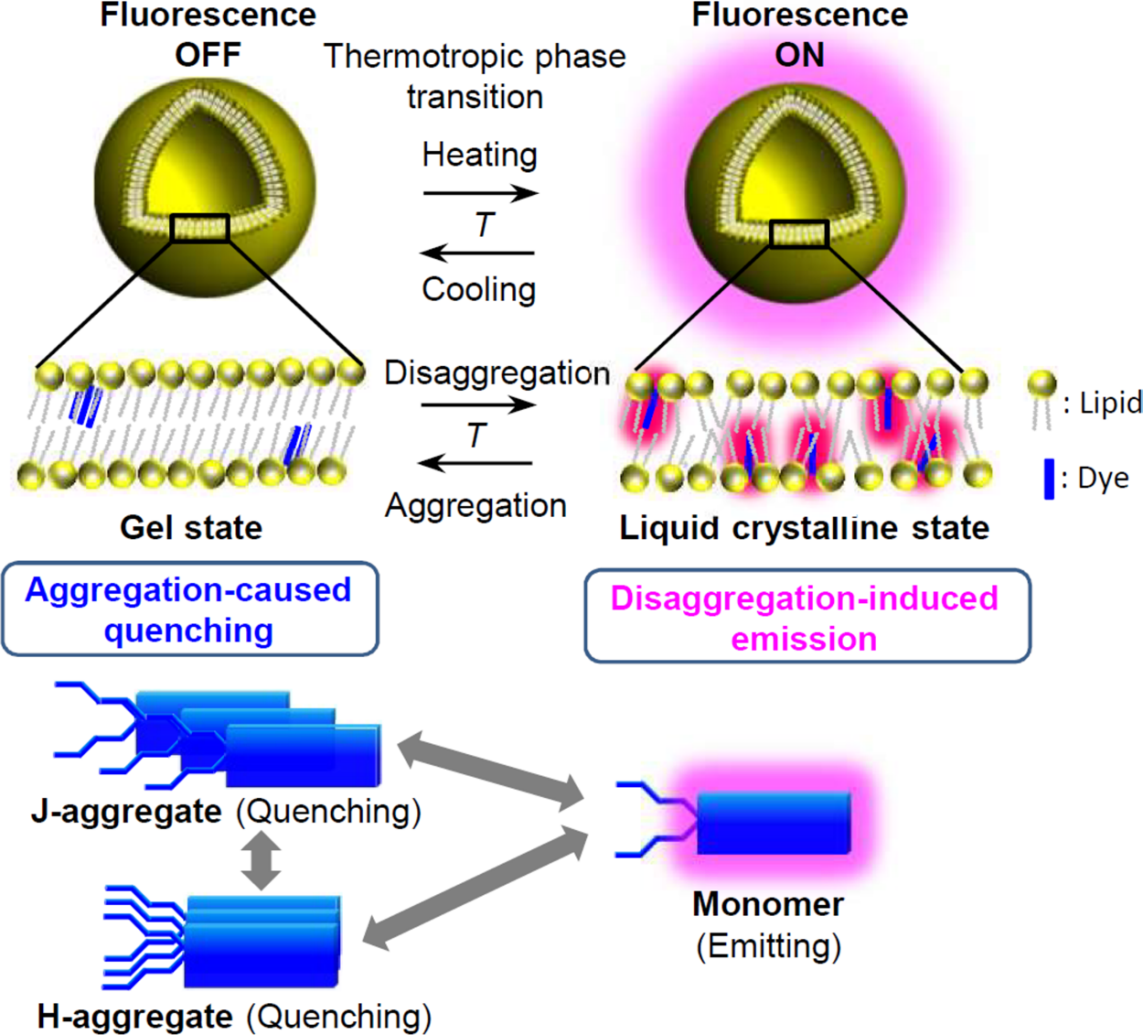
Proposed mechanism for SQR22 fluorescence switching in the lipid bilayer membrane as a response to phase transition. The fluorescence off/on switching is a result of the reversible aggregation-caused quenching/disaggregation-induced emission of SQR22 in the gel to liquid crystalline phase transition temperature (*T*) of the lipid bilayer membrane.

Based on the experimental observations, a fluorescence switching mechanism was proposed (Fig. 8). At 3.3 mol%, the SQR22 fluorescence of is quenched due to aggregation (H- and J-aggregates including dimer) in the lipid bilayer membrane at the solid gel. When the phase transitions to the liquid crystalline state, above the phase transition temperature, disaggregation occurs and fluorescence is emitted. The reversed phase transition from liquid crystalline phase to gel phase upon cooling is regarded as a crystallization of acyl chains of lipids. The exclusion of the dye from the crystalized acyl chains would facilitate the rapid formation of the dye aggregate in the lipid membrane at gel phase. This may be similar to the phase separation system of lipids with different acyl chain lengths having different phase transition temperature^47^. Such mechanism, based on self-assembly, would facilitate the development of threshold temperature sensors with remarkably high sensitivity. The results evidence the importance of further studies to apply phase transition phenomena for the measurement of fluctuating absolute temperatures. To read absolute temperatures from fluorescence intensity, there should be one-to-one correspondence between intensity and temperature, independent of heating and cooling. The present system would likely require a complicate analysis because of the hysteresis upon heating and cooling. The temperature hysteresis between transition pathways in heating and cooling processes has been discussed in relation to the occurrence of metastable ripple or ripple-gel mixed phases^48^. Therefore, future studies will consider a limited temperature range that does not cross the metastable ripple phase or the selection of different lipid species to minimize the temperature hysteresis and thus achieve a precise and stable thermometry for fluctuating absolute temperature. Temperature hysteresis is also observed in the phase transition or phase separation behavior of thermo-responsive polymers^49,50^ and the holding and unholding of DNA^51^. Hence, it should be noted that the two-way thermal characteristics of the thermosensitive matrix have to be considered to achieve a precise calibration curve to convert the fluorescence signal into temperature values. Our current study has achieved the sensing and imaging of threshold temperatures corresponding to phase transitions of nanovesicles. By using these fluorescent nanoprobes, for example, monitoring the threshold temperature and controlling the temperature range at the nano- and microscale for heat-triggered drug release from the nanovesicles^26,28,29^, digital polymerase chain reaction in microdroplets^52^ and protein expression in artificial cells or protocells^53^ become possible.

## Conclusion

Controlling molecular self-assembly of lipids and a fluorescent dye by phase transition leads to fluorescence switching at a critical transition temperature. The present study indicated that the fluorescence switching is induced by the reversible aggregation-caused quenching and disaggregation-induced emission of the dye in lipid bilayer membrane at the phase transition temperature. This mechanism provides a strategy beyond the function of fluorescence molecule to create a series of fluorescent nanomaterials with tunable fluorescence characteristics due to their high sensitivity to temperature. The sensitive to temperature in the present thermosensitive nanovesicles containing SQR22 reached to 22%/°C which is significantly higher than those of conventional thermosensitive fluorescent dyes (2–4%/°C). The findings of this work provide the basis for the development of advanced nanoprobes for optical temperature sensing and imaging in a wide number of fields, including chemistry, materials sciences, biology, and nanotechnology.

## Methods

### Materials

1,2-Dipalmitoyl-*sn*-glycero-3-phosphocholine (PC_16_), L-glutamic acid, *N*-(3-carboxy-1-oxopropyl)-, and 1,5-dihexadecyl ester (SA) were purchased from Nippon Fine Chemical Co. Ltd. (Osaka, Japan). 1,2-Dimyristoyl-*sn*-glycero-3-phosphocholine (PC_14_), 1,2-dipentadecanoyl-*sn*-glycero-3-phosphocholine (PC_15_), 1,2-diheptadecanoyl-*sn*-glycero-3-phosphocholine (PC_17_), and 1,2-distearoyl-*sn*-glycero-3-phosphocholine (PC_18_) were acquired from Avanti Polar Lipids Inc. (Alabaster, AL, USA). 1,2-Distearoyl-*sn*-glycero-3-phosphoethanolamine-*N*-[monomethoxy poly(ethylene glycol) (5000)] (PEG-DSPE) was purchased from NOF Corp. (Tokyo, Japan).

### Synthesis and characterization

The squaraine dye, SQR22, including three fragments: aniline, squaric core, and indolium was designed and synthesized. The chemical structure of the obtained compounds was confirmed by ^1^H and ^13^C NMR spectroscopy (CDCl_3_, 400 MHz) and mass spectrometry. Details of the synthesis and characterization of the SQR22 are provided in the Supporting Information File (Figs. S1–S3).

### Spectroscopic properties of SQR22

For the spectroscopy experiments, SQR22 was dissolved in various solvents at 10 µM. It was not possible to disperse solid SQR22 directly in water; thus, an ethanol stock solution (2.26 mM) of the dye was injected in water. The fluorescence of the SQR22 solutions was observed on a transilluminator (LAS-1000 UV mini; Fujifilm, Tokyo, Japan) at 365 nm. The UV-vis-near infrared spectra were measured using a UV-vis spectrophotometer (V-670; JASCO Co., Tokyo, Japan). The fluorescence excitation and emission spectra were recorded with a fluorescence spectrophotometer (Agilent Cary Eclipse; Agilent Technologies, Santa Clara, USA). The fluorescence quantum yields of SQR22 were calculated using Eq. (1).

1 $$\varPhi ={\varPhi }_{st}\cdot \frac{{A}_{st}}{A}\cdot \frac{I}{{I}_{st}}$$

where *Φ*, *A*, and *I* are the fluorescence quantum yield, absorbance and integral fluorescence intensity of the samples, and *Φ*_*st*_, *A*_*st*_, and *I*_*st*_ are the parameters for the reference, Rhodamine B, (*Φ*_*st*_ = 0.69 in ethanol, excitation at 366 nm)^54^.

#### Preparation and characterization of lipid nanovesicles containing SQR22

Nanovesicle samples were prepared using PC (PC_14_, PC_15_, PC_16_, PC_17_, or PC_18_), SA, and PEG-DSPE with a PC/SA/PEG-DSPE molar ratio of 9/1/0.06. The PC/SA/PEG-DSPE (9/1/0.06, molar ratio) mixed lipid powder with 0.1–3.3 mol% SQR22 were dissolved in *t*-butyl alcohol, then freeze-dried to obtain a mixed lipid powder with SQR22. This powder was hydrated with PBS solution ([lipids] = 5 mg/mL). Then, the dispersion was extruded (Northern Lipids Inc. Extruder, Canada) through membrane filters (0.05 µm final pore size, Isopore; Millipore Corp., Tokyo, Japan) above the phase transition temperature of PC under pressure using nitrogen gas. The diameters of the resulting liposomes were measured using a particle size analyzer based on dynamic light scattering (Zeta-Sizer Nano ZS; Malvern Instruments, Ltd., Malvern, Worcestershire, UK). The average diameter ± SD was calculated. The concentration of SQR22 in each liposome dispersion was determined based on the absorbance at 631 nm in ethanol.

#### Thermosensitive fluorescence properties of PC-nanovesicles with SQR22

The fluorescence intensity of the SQR22 lipid nanoparticles dispersion was measured using a spectrophotometer (λ_ex_ = 570 nm, λ_em_ = 660 nm, F-2700; Hitachi Ltd., Tokyo, Japan) at various temperatures while cooling. A spectrophotometer (Agilent Cary Eclipse; Agilent Technologies Inc., Santa Clara, USA) equipped with a water Peltier system (PCB 1500; Agilent Technologies Inc., Santa Clara, USA) was used for the fluorescence measurement while heating. The fluorescence intensity (percentage based on the maximum value) was plotted as a function of temperature. The UV-vis-near infrared spectra were measured using a UV-vis spectrophotometer (V-670; Jasco Corp., Tokyo, Japan).

#### Microscopic observation of fluorescence switching

The PC_16_/SA/PEG-DSPE (9/1/0.06, molar ratio) mixed lipid powder with 3.3 mol% SQR22 was gently hydrated with PBS ([lipids] = 5 mg/mL) at 50 °C. Agarose (3%) was dissolved in PBS at 80 °C and cooled to around 25 °C. Then, the dispersion of multilamellar vesicles was added to the agarose solution ([SQR22] = 5 μM). The mixed solution was added dropwise on a 35-mm glass bottom dish and allowed to solidify in a refrigerator (gel point; 8–17 °C). All imaging experiments were conducted with a confocal laser microscope (FV1000 IX81; Olympus Corp.) with an oil immersion lens (PLAPO, 60 × 1.45 NA). A 633 nm laser was used for excitation to observe the SQR22 multilamellar vesicles; fluorescence emission was measured from 645 to 745 nm. Images were recorded every 0.564 s. To heat the gel sample, a 980 nm continuous wave laser (700 mW) was used with a collimating lens (5-mm-diameter spot size). The temperature was maintained at 33 °C during microscopic observation using a temperature control chamber.

## Supplementary information

Supplementary information

## References

[1] Menges F (2016). Temperature mapping of operating nanoscale devices by scanning probe thermometry. Nat. Commun..

[2] Ross D, Gaitan M, Locascio LE (2001). Temperature measurement in microfluidic systems using a temperature-dependent fluorescent dye. Anal. Chem..

[3] Brites CDS (2012). Thermometry at the nanoscale. Nanoscale.

[4] Arai, S. & Suzuki, M. Nanosized optical thermometers. In *Smart nanoparticles for biomedicine* (ed. Ciofani, G.) Chapter 14, 199–217 (Elsevier, 2018).

[5] Geitenbeek RG (2019). Luminescence thermometry for *in situ* temperature measurements in microfluidic devices. Lab Chip.

[6] Suzuki M, Tseeb V, Oyama K, Ishiwata S (2007). Microscopic detection of thermogenesis in a single HeLa cell. Biophys. J..

[7] Arai S, Lee SC, Zhai D, Suzuki M, Chang YT (2014). A molecular fluorescent probe for targeted visualization of temperature at the endoplasmic reticulum. Sci. Rep..

[8] Arai S (2015). Mitochondria-targeted fluorescent thermometer monitors intracellular temperature gradient. Chem. Commun..

[9] Donner JS, Thompson SA, Kreuzer MP, Baffou G, Quidant R (2012). Mapping intracellular temperature using green fluorescent protein. Nano Lett..

[10] Fischer LH, Harms GS, Wolfbeis OS (2011). Upconverting nanoparticles for nanoscale thermometry. Angew. Chemie - Int. Ed..

[11] Ye F (2011). Ratiometric temperature sensing with semiconducting polymer dots. J. Am. Chem. Soc..

[12] Yang JM, Yang H, Lin L (2011). Quantum dot nano thermometers reveal heterogeneous local thermogenesis in living cells. ACS Nano.

[13] Kucsko G (2013). Nanometre-scale thermometry in a living cell. Nature.

[14] Shang L, Stockmar F, Azadfar N, Nienhaus GU (2013). Intracellular thermometry by using fluorescent gold nanoclusters. Angew. Chemie - Int. Ed..

[15] Oyama K (2012). Walking nanothermometers: Spatiotemporal temperature measurement of transported acidic organelles in single living cells. Lab Chip.

[16] Takei Y (2014). A nanoparticle-based ratiometric and self-calibrated fluorescent thermometer for single living cells. ACS Nano.

[17] Miyagawa T (2016). Glue-free stacked luminescent nanosheets enable high-resolution ratiometric temperature mapping in living small animals. ACS Appl. Mater. Interfaces.

[18] Uchiyama S, Matsumura Y, De Silva AP, Iwai K (2003). Fluorescent molecular thermometers based on polymers showing temperature-induced phase transitions and labeled with polarity-responsive benzofurazans. Anal. Chem..

[19] Okabe K (2012). Intracellular temperature mapping with a fluorescent polymeric thermometer and fluorescence lifetime imaging microscopy. Nat. Commun..

[20] Cui J, Kwon JE, Kim HJ, Whang DR, Park SY (2017). Smart fluorescent nanoparticles in water showing temperature-dependent ratiometric fluorescence color change. ACS Appl. Mater. Interfaces.

[21] Ke G (2012). L-DNA molecular beacon: a safe, stable, and accurate intracellular nano-thermometer for temperature sensing in living cells. J. Am. Chem. Soc..

[22] Gareau D, Desrosiers A, Vallée-Bélisle A (2016). Programmable quantitative DNA nanothermometers. Nano Lett..

[23] Torchilin VP (2005). Recent advances with liposomes as pharmaceutical carriers. Nat. Rev. Drug Discov..

[24] Koynova R, Caffrey M (1998). Phases and phase transitions of the phosphatidylcholines. Biochim. Biophys. Acta - Rev. Biomembr..

[25] Al-Ahmady Z, Kostarelos K (2016). Chemical components for the design of temperature-responsive vesicles as cancer therapeutics. Chem. Rev..

[26] Arai S, Lee CLK, Chang YT, Sato H, Sou K (2015). Thermosensitive nanoplatforms for photothermal release of cargo from liposomes under intracellular temperature monitoring. RSC Adv..

[27] Sou K, Chan LY, Lee CLK (2016). Temperature tracking in a three-dimensional matrix using thermosensitive liposome platform. ACS Sens..

[28] Le DL (2018). Neurotransmitter-loaded nanocapsule triggers on-demand muscle relaxation in living organism. ACS Appl. Mater. Interfaces.

[29] Sou K, Le DL, Sato H (2019). Nanocapsules for Programmed neurotransmitter release: toward artificial extracellular synaptic vesicles. Small.

[30] Liu X (2015). Development of asymmetrical near infrared squaraines with large stokes shift. RSC Adv..

[31] Shafeekh KM, Das S, Sissa C, Painelli A (2013). Asymmetric squaraine dyes: Spectroscopic and theoretical investigation. J. Phys. Chem. B.

[32] Marini A, Munnoz-Losa A, Biancardi A, Mennucci B (2010). What is solvatochromism?. J. Phys. Chem. B.

[33] Kao YJ, Soutar AK, Hong KY, Pownall HJ, Smith LC (1978). N-(2-naphthyl)-23,24-dinor-5-cholen-22-amin-β-ol, a fluorescent cholesterol analogue. Biochemistry.

[34] Jiang N (2015). Ratiometric fluorescence imaging of cellular polarity: decrease in mitochondrial polarity in cancer cells. Angew. Chemie - Int. Ed..

[35] Eisfeld A, Briggs JS (2006). The J-and H-bands of organic dye aggregates. Chem. Phys..

[36] Chen C (2011). A squaraine-based colorimetric and “turn on” fluorescent sensor for selective detection of Hg2+ in an aqueous medium. Org. Lett..

[37] Li M, Niu Y, Lu HY, Chen CF (2015). Tetrahydro [5] helicene-based dye with remarkable and reversible acid/base stimulated fluorescence switching properties in solution and solid state. Dyes and Pigments.

[38] Klibanov AL, Maruyama K, Torchilin VP, Huang L (1990). Amphipathic polyethyleneglycols effectively prolong the circulation time of liposomes. FEBS Lett..

[39] Sou K (2011). Electrostatics of carboxylated anionic vesicles for improving entrapment capacity. Chem. Phys. Lipids.

[40] Kinnunen PKJ (2009). Amyloid formation on lipid membrane surfaces. Open Biol..

[41] Bui TT, Suga K, Umakoshi H (2016). Roles of sterol derivatives in regulating the properties of phospholipid bilayer systems. Langmuir.

[42] Arias JM, Tuttolomondo ME, Díaz SB, Ben Altabef A (2018). Reorganization of hydration water of DPPC multilamellar vesicles induced by l-cysteine interaction. J. Phys. Chem. B.

[43] Neupane S, De Smet Y, Renner FU, Losada-Perez P (2018). Quartz crystal microbalance with dissipation monitoring: a versatile tool to monitor phase transitions in biomimetic membranes. Front. Mater..

[44] Sun L, Böckmann RA (2018). Membrane phase transition during heating and cooling: molecular insight into reversible melting. Eur. Biophys. J..

[45] Dong S, Teo JDW, Chan LY, Lee C-LK, Sou K (2018). Far-red fluorescent liposomes for folate receptor-targeted bioimaging. ACS Appl. Nano Mater..

[46] Chen H, Law K, Whitten DG (1996). Aggregation of amphiphilic squaraines at the air - water interface and in langmuir - blodgett films. J. Phys. Chem..

[47] Ehrig J, Petrov EP, Schwille P (2011). Phase separation and near-critical fluctuations in two-component lipid membranes: Monte Carlo simulations on experimentally relevant scales. New J. Phys..

[48] Tenchov B (1991). On the reversibility of the phase transitions in lipid-water systems. Chem. Phys. Lipids.

[49] Ray B (2005). Effect of tacticity of poly (N-isopropylacrylamide) on the phase separation temperature of its aqueous solutions. Polym. J..

[50] Seuring J, Agarwal S (2010). Non-ionic homo- and copolymers with H-donor and H-acceptor units with an UCST in water. Macromol. Chem. Phys..

[51] Kankia B (2016). Stable domain assembly of a monomolecular DNA quadruplex: Implications for DNA-based nanoswitches. Biophys. J..

[52] Sreejith KR, Ooi CH, Jin J, Dao DV, Nguyen NT (2018). Digital polymerase chain reaction technology - recent advances and future perspectives. Lab Chip.

[53] Jia H, Heymann M, Härtel T, Kai L, Schwille P (2019). Temperature-sensitive protein expression in protocells. Chem. Commun..

[54] Parker CA, Rees WT (1960). Correction of fluorescence spectra and measurement of fluorescence quantum efficiency. Analyst.

