# Supplementary material for: Highly cooperative fluorescence switching of self-assembled squaraine dye at tunable threshold temperatures using thermosensitive nanovesicles for optical sensing and imaging
Source: Sci Rep. 2019 Nov 29;9:17991. doi: 10.1038/s41598-019-54418-1 (PMC6884458; doi:10.1038/s41598-019-54418-1)
Supplement: Supplementary file 1 — Supplementary information [file 41598_2019_54418_MOESM1_ESM.pdf]

Supplementary Information

Highly cooperative fluorescence switching of self-assembled squaraine dye at tunable threshold temperatures using thermosensitive nanovesicles for optical sensing and imaging

Keitaro Sou*, Li Yan Chan, Satoshi Arai, Chi-Lik Ken Lee

* Corresponding author. Email: soukei@aoni.waseda.jp (K.S.)

Table of contents:

1. Synthesis and characterization of SQR22.

Figure S1. Synthesis of SQR22.

Figure S2. ^{1}H NMR spectra of SQR22 ($CDCl_3$, 400 MHz).

Figure S3. ^{13}C NMR spectra of SQR22 ($CDCl_3$, 400 MHz).

2. Spectroscopic properties of SQR22.

Table S1. Spectroscopic properties of SQR22.

3. Characterization of lipid nanovesicles containing SQR22 (PC-NVSQ).

Table S2. Characteristics of lipid nanovesicles containing SQR22 (PC-NVSQ).

4. Repeatability of fluorescence switching.

Figure S4. Repeatability of fluorescence switching for PC_{14}-NVSQ, PC_{15}-NVSQ, PC_{17}-NVSQ, and PC_{18}-NVSQ ([SQR22] = 1 μM) through 10 heating/cooling cycles.

5. Temperature-dependent fluorescence and UV-vis-NIR spectra of PC_{16}-NV containing SQR22.

Figure S5. Change in fluorescence emission spectra as a function of temperature in PC_{16}-NV containing 3.3 mol% SQR22 (PC_{16}-NVSQ).

Figure S6. UV-vis-NIR spectra of PC_{16}-NV containing SQR22.

1. Synthesis and characterization of SQR22.

Synthesis of SQR22. The synthesis of squaraine dye, SQR22, is outlined in **Figure S1**. The design includes three fragments: aniline, squaric core, and indolium. The sequence used for preparing the semi-squaraine intermediate is crucially important to form the asymmetrical squaraine dyes. Precursors *N,N*-dibutylaniline **1** and squaryl chloride **2** were prepared based on earlier reported procedures.[S1] Coupling of **1** and **2** under refluxing toluene gave the chloride intermediate, which was then hydrolyzed in a one-pot fashion to obtain squaric acid **3**. Indolium salt **4** was synthesized using earlier published procedures.[S2] Reacting **3** and **4** together in a 1:1 mixture of refluxing *n*-butanol/toluene yielded the butyl ester squaraine dye **5** because the expected carboxylic group of the squaraine dyes reacted with the *n*-butanol solvent to form the butyl ester derivatives instead. We originally designed the squaraine to contain the carboxylic group because this is expected to serve as an important design for further functionalization and application. However, when **5** was subjected to base hydrolysis using Na^{t}OBu in THF at room temperature, SQR22 was obtained exclusively instead of the butyl ester deprotected **5**.

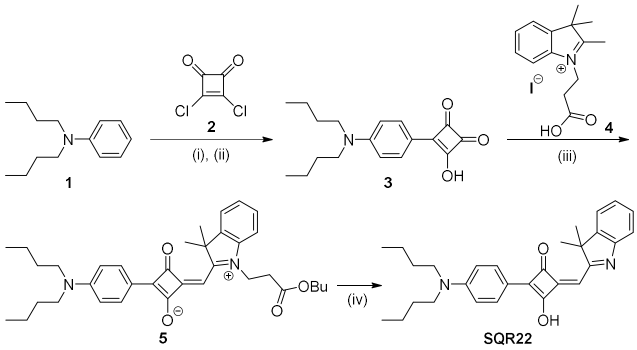

Figure S1. Synthesis of SQR22. Reagents and conditions: (i) toluene, reflux, 6 h; (ii) 5N HCl, AcOH/H_2O (1:1), reflux, 2 h; (iii) *n*-BuOH/toluene (4:1), reflux, 3 h; (iv) Na^{t}OBu, THF, rt, 3 h.

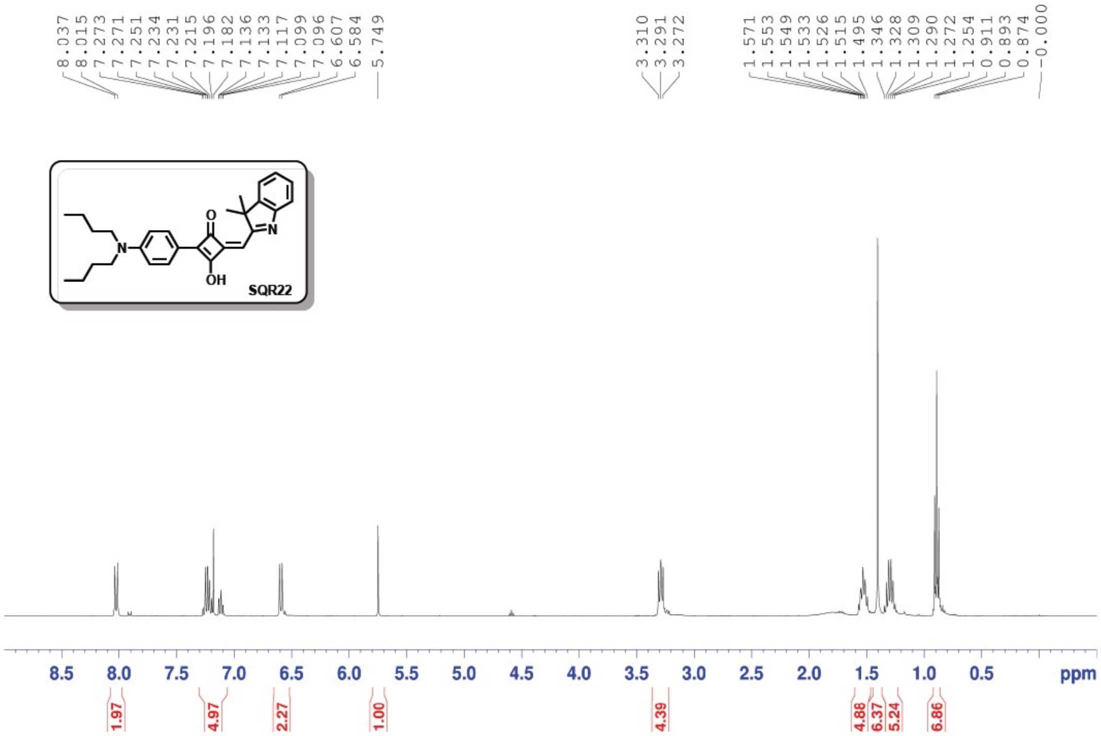

Figure S2. ^{1}H NMR spectra of SQR22 ($CDCl_3$, 400 MHz).

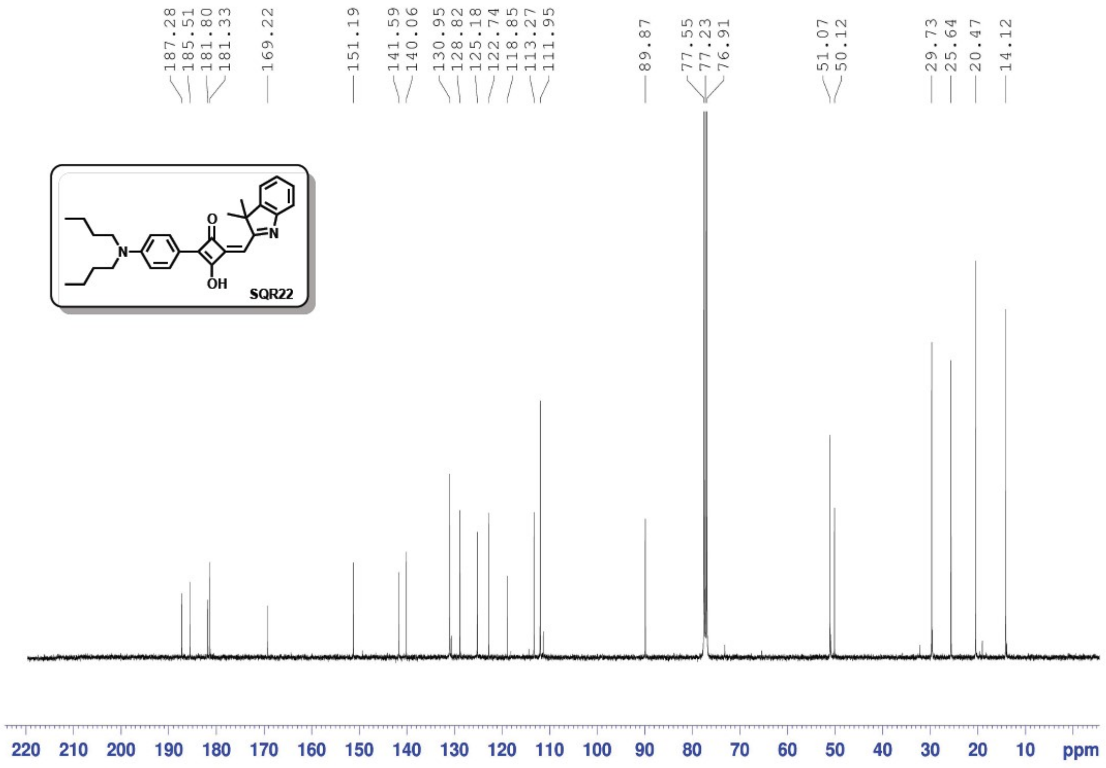

Figure S3. ^{13}C NMR spectra of SQR22 ($CDCl_3$, 400 MHz).

Characterization of SQR22.

^{1}H NMR ($CDCl_3$, 400 MHz, δ ppm): 0.89 (t, J = 7.3 Hz, 6H, $-CH_3$); 1.25–1.35 (m, 4H, $-CH_2-$); 1.41 (s, 6H, $-CH_3$); 1.50–1.57 (m, 4H, $-CH_2-$); 3.27–3.31 (m, 4H, $-CH_2-$); 5.75 (s, 1H, =CH-); 6.60 (d, J = 9.2 Hz, 2H, ArH), 7.04–7.36 (m, 4H, ArH); 8.03 (d, J = 9.1 Hz, 2H, ArH). ^{13}C NMR ($CDCl_3$, 400 MHz, δ ppm): 14.12, 20.47, 25.64, 29.61, 50.12, 51.07, 89.87, 111.95, 113.27, 118.85, 122.74, 125.18, 128.82, 130.95, 140.06, 141.59, 151.19, 169.22, 181.33, 181.80, 185.51, 187.28. MS (ESI) *m*/*z* (M^+) 443.

2. Spectroscopic Properties of SQR22.

For the spectroscopy experiments, SQR22 was dissolved in various solvents at 10 μM. It was not possible to disperse solid SQR22 directly in water; thus, an ethanol stock solution (2.26 mM) of the dye was injected in water. The fluorescence of the SQR22 solutions was observed on a transilluminator (LAS-1000 UV mini; Fujifilm, Tokyo, Japan) at 365 nm. The UV-vis-near infrared spectra were measured using a UV-vis spectrophotometer (V-670; JASCO Co., Tokyo, Japan). The fluorescence excitation and emission spectra were recorded with a fluorescence spectrophotometer (Agilent Cary Eclipse; Agilent Technologies, Santa Clara, USA). The fluorescence quantum yields of SQR22 were calculated using equation (1).

$$\Phi = \Phi_{st} \cdot \frac{A_{st}}{A} \cdot \frac{I}{I_{st}} \quad (1)$$

where Φ, A, and I are the fluorescence quantum yield, absorbance and integral fluorescence intensity of the samples, and Φ_{st}, A_{st}, and I_{st} are the parameters for the reference, Rhodamine B, (Φ_{st} = 0.69 in ethanol, excitation at 366 nm).[S3]

Table S1 Spectroscopic properties of SQR22 in various solvents

No	Solvent	Polarity index	Dielectric constant	λ_{max} (nm)	λ_{em} (nm)	Quantum yield
1	Hexane	0.1	1.9	629	640	0.44
2	Cyclohexane	0.2	2.0	633	642	0.40
3	Toluene	2.4	2.4	636	655	0.36
4	Benzene	2.7	2.3	637	656	0.36
5	Dichloromethane	3.1	9.1	633	659	0.38
6	THF	4.0	7.6	633	660	0.42
7	Chloroform	4.1	4.8	635	656	0.40
8	Ethanol	4.3	24.6	631	662	0.32
9	Methanol	5.1	32.6	628	662	0.13
10	Acetone	5.1	20.7	629	663	0.23
11	Acetonitrile	5.8	37.5	626	663	0.22
12	DMF	6.4	36.7	474	552	0.02
13	DMSO	7.2	47	477	565	0.02
14	Water	10.2	78.5	595 (broad)	non	0

3. Characterization of lipid nanovesicles containing SQR22 (PC-NVSQ).

Characteristics of lipid nanovesicles containing SQR22 are summarized in Table S2.

Table S2. Characteristics of lipid nanovesicles containing SQR22 (PC-NVSQ)

Samples	Lipid compositions (molar ratio)	Lipids/SQR22 (w/w)	SQR22 (mol%)	Size (nm)	λ_{max} a) (nm)	λ_{em} b) (nm)	T c) (°C)
PC_{14}-NVSQ	PC_{14}/SA/PEG-DSPE (9/1/0.06)	50	3.1	104 ± 25	625	664	24
PC_{15}-NVSQ	PC_{15}/SA/PEG-DSPE (9/1/0.06)	50	3.2	106 ± 29	631	664	35
PC_{16}-NVSQ	PC_{16}/SA/PEG-DSPE (9/1/0.06)	50	3.3	105 ± 25	636	664	40
PC_{17}-NVSQ	PC_{17}/SA/PEG-DSPE (9/1/0.06)	50	3.4	106 ± 26	637	664	49
PC_{18}-NVSQ	PC_{18}/SA/PEG-DSPE (9/1/0.06)	50	3.5	111 ± 27	636	662	54

a) UV-vis-NIR absorbance spectra were measured at 25 °C. b) Fluorescence emission spectra were measured at 40, 45, 50, 55, and 60 °C, respectively, for PC_{14}-, PC_{15}-, PC_{16}-, PC_{17}-, and PC_{18}-NVSQ. c) Phase transition temperatures (T) were obtained from previous reports for PC_{14} and PC_{18},[S4] PC_{16},[S5] and PC_{15} and PC_{17}.[S6]

4. Repeatability of fluorescence switching.

Fluorescence intensity of the nanovesicle dispersion containing SQR22 (PC-NVSQ) was measured using a fluorescence spectrophotometer (λ_{ex}=570 nm, λ_{em}=660 nm, F-2700; Hitachi Ltd., Tokyo, Japan). The temperatures at heating were 39, 42, 48, 54, and 60 °C, respectively, for PC_{14}-, PC_{15}-, PC_{16}-, PC_{17}-, and PC_{18}-NVSQ. The fluorescence intensity at cooling was measured at 25 °C for all samples. The result for PC_{16}-NVSQ is shown in **Figure 3c**.

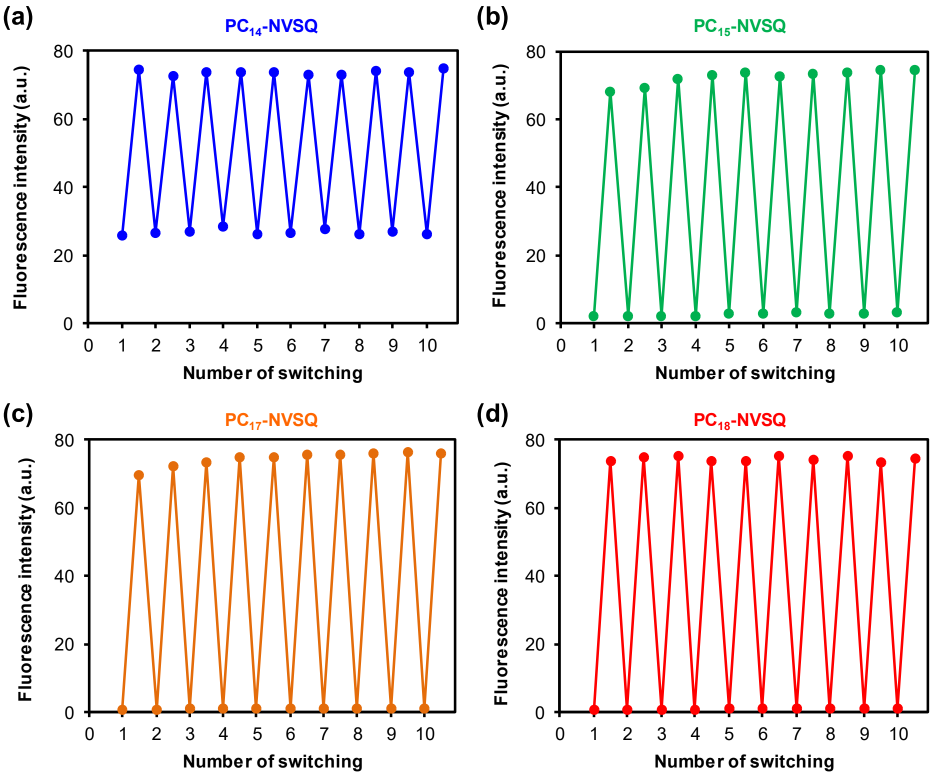

Figure S4. Repeatability of fluorescence switching for (a) PC_{14}-NVSQ, (b) PC_{15}-NVSQ, (c) PC_{17}-NVSQ, and (d) PC_{18}-NVSQ ([SQR22] = 1 μM) through 10 heating/cooling cycles.

5. Temperature-dependent fluorescence and UV-vis-NIR spectra of PC16-NV containing SQR22.

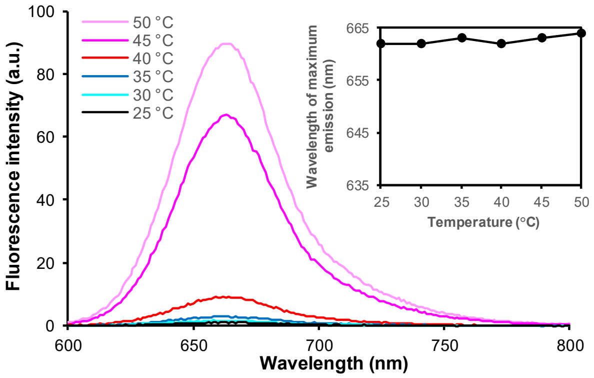

Figure S5. Change in fluorescence emission spectra as a function of temperature in PC_{16}-NV containing 3.3 mol% SQR22 (PC_{16}-NVSQ). The insertion shows the wavelength of maximum fluorescence intensity at each temperature.

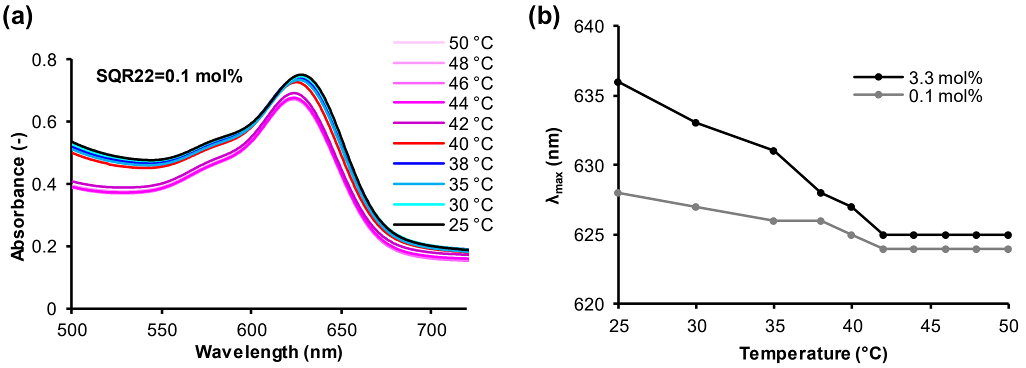

Figure S6. UV-vis-NIR spectra of PC_{16}-NV containing SQR22. (a) Change in UV-vis-NIR spectra as a function of temperature in PC_{16}-NV containing 0.1 mol% SQR22 ([SQR22] = 10 µM). (b) Comparison of maximum absorption wavelength (λ_{max}) change as a function of temperature between PC_{16}-NV containing 3.3 and 0.1 mol% SQR22 ([SQR22] = 10 µM).

References

[S1] (a) F. Li, N. Gao, H. Xu, W. Liu, H. Shang, W. Yang, M. Zhang, *Chem. Eur. J.* **2014**, *20*, 9991; (b) A. Easwaran, C. Parayali, A. Ayyappanpillai, *J. Am. Chem. Soc.* **2004**, *126*, 6590.

[S2] D. Liu, W. Chen, K. Sun, K. Deng, W. Zhang, Z. Wang, X. Jiang, *Angew. Chem. Int. Ed.* **2011**, *50*, 4103.

[S3] C. A. Parker, W. T. Rees, *Analyst* **1960**, *85*, 587.

[S4] R. Koynova, M. Caffrey, M. *Biochim. Biophys. Acta - Rev. Biomembr.* **1998**, *1376*, 91.

[S5] S. Arai, C.-L. K. Lee, Y.-T. Chang, H. Sato, K. Sou, *RSC Adv.* **2015**, *5*, 93530.

[S6] K. Sou, L. Y. Chan, C.-L. K. Lee, *ACS Sens.* **2016**, *1*, 650.
